# *C9orf72* intermediate expansions of 24–30 repeats are associated with ALS

**DOI:** 10.1186/s40478-019-0724-4

**Published:** 2019-07-17

**Authors:** Alfredo Iacoangeli, Ahmad Al Khleifat, Ashley R. Jones, William Sproviero, Aleksey Shatunov, Sarah Opie-Martin, Alzheimer’s Disease Neuroimaging Initiative, Karen E. Morrison, Pamela J. Shaw, Christopher E. Shaw, Isabella Fogh, Richard J. Dobson, Stephen J. Newhouse, Ammar Al-Chalabi

**Affiliations:** 1 Department of Biostatistics and Health Informatics, King’s College London, London, UK; 2 Department of Basic and Clinical Neuroscience, King’s College London, Maurice Wohl Clinical Neuroscience Institute, London, UK; 3 The Alzheimer’s Disease Neuroimaging Initiative, Center for Imaging of Neurodegenerative Disease, San Francisco VA Medical Center, University of California, San Francisco, USA; 4 Faculty of Medicine, University of Southampton, University Hospital Southampton, Southampton, UK; 5 Sheffield Institute for Translational Neuroscience, University of Sheffield, Sheffield, UK; 6 UK Dementia Research Institute, King’s College London, London, UK; 7 Department of Neurology and Laboratory of Neuroscience, IRCCS Istituto Auxologico Italiano, Milan, Italy; 8 Farr Institute of Health Informatics Research, UCL Institute of Health Informatics, University College London, London, UK; 9 National Institute for Health Research (NIHR) Biomedical Research Centre and Dementia Unit at South London and Maudsley NHS Foundation Trust and King’s College London, London, UK; 10 King’s College Hospital, Bessemer Road, London, SE5 9RS UK

**Keywords:** *C9orf72*, Repeat expansion, ALS, Genetics, Whole-genome sequencing, Next-generation sequencing

## Abstract

The expansion of a hexanucleotide repeat GGGGCC in C9orf72 is the most common known cause of ALS accounting for ~ 40% familial cases and ~ 7% sporadic cases in the European population. In most people, the repeat length is 2, but in people with ALS, hundreds to thousands of repeats may be observed. A small proportion of people have an intermediate expansion, of the order of 20 to 30 repeats in size, and it remains unknown whether intermediate expansions confer risk of ALS in the same way that massive expansions do. We investigated the association of this intermediate repeat with ALS by performing a meta-analysis of four previously published studies and a new British/Alzheimer’s Disease Neuroimaging Initiative dataset of 1295 cases and 613 controls. The final dataset comprised 5071 cases and 3747 controls. Our meta-analysis showed association between ALS and intermediate C9orf72 repeats of 24 to 30 repeats in size (random-effects model OR = 4.2, 95% CI = 1.23–14.35, *p*-value = 0.02). Furthermore, we showed a different frequency of the repeat between the northern and southern European populations (Fisher’s exact test *p*-value = 5 × 10^− 3^). Our findings provide evidence for the association between intermediate repeats and ALS (*p*-value = 2 × 10^− 4^) with direct relevance for research and clinical practice by showing that an expansion of 24 or more repeats should be considered pathogenic.

**Electronic supplementary material:**

The online version of this article (10.1186/s40478-019-0724-4) contains supplementary material, which is available to authorized users.

## Introduction

Amyotrophic lateral sclerosis (ALS) is a neurodegenerative disease, primarily affecting upper and lower motor neurons, resulting in progressive weakness and culminating in death from neuromuscular respiratory failure, typically 2–5 years after diagnosis [1]. The incidence is 1–2 per 100,000 person-years, point prevalence is 3 to 5 per 100,000 people in Europe and the United States, and the lifetime risk is 1 in 300 [2–4].

Following initial linkage and association studies, the massive expansion of a hexanucleotide repeat in the *C9orf72* gene was found to be the most frequent cause of ALS [5–10]. In most people, the repeat length is 2, but in people with ALS, hundreds to thousands of repeats may be observed [11]. A small proportion of people have an intermediate expansion, of the order of 20 to 30 repeats in size and several studies suggest that a threshold of 20 or 23 repeats could be used to discriminate between pathogenic and neutral expansions [7, 11–16]. However, the rarity of intermediate repeats and the consequential lack of statistical power, has limited the validity of these results and it remains unknown whether intermediate expansions confer risk of ALS in the same way that massive expansions do. At present, a repeat length of > 30 is typically used as the threshold to distinguish between neutral and pathogenic expansions [5].

One meta-analysis based on four studies, suggested an association of intermediate expansions between 24 and 30 repeats in size with sporadic ALS (chi-squared *p*-value = 0.03, fixed-effect model) [17]. However, while this meta-analysis was rigorous, it had a few limitations which prevented the results translating into research and medical practice. First, several studies were excluded because data on repeats of length < 30 were not available [17]. Second, the fixed-effect model used in the meta-analysis assumes that the genetic effects are the same across the combined investigations, and that all differences are due to chance [18, 19]. While this assumption appears to be supported by the lack of heterogeneity across the involved studies (*Q*-test *p*-value = 0.61), heterogeneity may be masked. Genetic effects may vary across different populations for various reasons, including both genuine differences and differential biases and errors across studies [20, 21], and the *C9orf72* repeat expansion exemplifies this variation [13, 15, 16, 22, 23]. Moreover, the *Q*-test and *I*-squared do not describe heterogeneity well when the number of studies is small [24, 25] and the datasets did not provide sufficient power to properly test the meta-analysis studies for heterogeneity. Third, other studies of intermediate repeat sizes have been inconclusive [5, 7, 14].

**Fig. 1:**
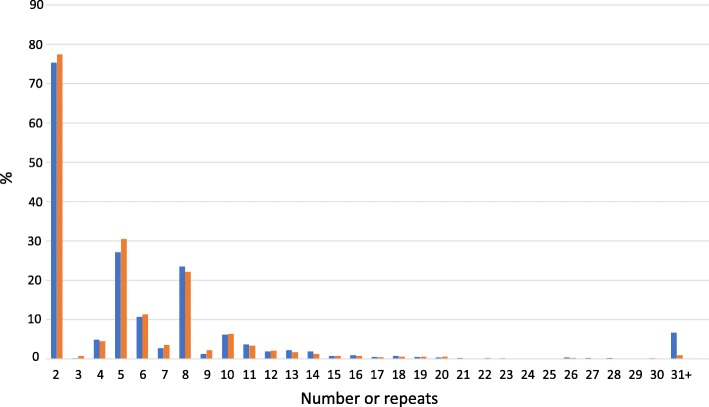
Distribution of the samples carrying at least one allele of N repeats (x axis) in our dataset (British/ADNI) for cases (blue) and controls (orange)

Taking these considerations into account, we therefore analysed a new group of 1295 people with ALS and 613 controls, all sized for *C9orf72* repeats, and included the findings in a meta-analysis of studies for which there are data on intermediate repeats of size 24 or greater. We did not investigate intermediate repeats of size 20 to 23 since such repeats have been observed both in ALS patients and controls with no differences in allele distribution in several studies [5, 16, 23], including in our new cohort (Fig. 1 and Additional file 1: **Table S1**).

## Methods

### Whole-genome sequencing samples

Whole-genome sequencing (WGS) data were obtained from blood samples of 1908 individuals, comprising 1295 people with ALS (all apparently sporadic cases) and 613 unaffected controls from two groups: a UK set of 1295 ALS cases, 340 matched controls [26], and 273 controls from the Alzheimer’s Disease Neuroimaging Initiative (ADNI) WGS dataset (http://adni.loni.usc.edu). The UK dataset was generated as part of Project MinE [26, 27], on the Illumina Hiseq X platform (150 bp paired-end reads) and the ADNI dataset on the Illumina 2000 platform (100 bp paired-end reads).

### Genotyping

We used ExpansionHunter [28] to size the *C9orf72* GGGGCC repeat in the WGS data. ExpansionHunter has been previously validated for *C9orf72* repeat sizing in 5787 WGS samples from Project MinE that were also genotyped using repeat-primed polymerase chain reaction (PCR) and fluorescence PCR. Using the repeat-primed PCR calls as the gold standard, ExpansionHunter showed an expansion detection accuracy > 99% [28]. ExpansionHunter also showed high concordance with PCR and Sanger sequencing in repeats whose total length did not exceed the read-length [28, 29], while providing a confidence interval for longer repeats. We used the ExpansionHunter repeat length called when repeat sizes had a read length < 25 repeats for the UK dataset and < 17 repeats for the ADNI dataset to account for the difference in sequencing platform, and we used the middle value of the confidence interval for longer repeats.

### Meta-analysis and statistics

For meta-analysis, we selected studies for which data were available for repeats of size 24 or more [17]. Association between the number of repeats and disease status was analysed by chi-square and Fisher’s exact tests, with the samples split into two categories: samples with an intermediate expansion of 24–30 repeats and the remainder including those with repeat length > 30. Heterogeneity across studies was examined using the *I*-squared statistic and chi-square-based *Q*-tests. The *Q*-test power was calculated using the Jackson method [25]. Both fixed-effect and random-effects models were used. Sensitivity analyses were conducted to evaluate the influence of individual results on the pooled estimate after sequential removal of each study. Potential publication bias was assessed by both Begg and Egger tests. The Kolmogorov-Smirnov test was used to assess the difference of the repeat length distributions between cases and controls. To test the phenotype differences between our intermediate repeat carriers (> 23 and < = 30), the expansion carriers (> 30) and the non-expanded patients (< 24), we used the logrank test to compare the survival distributions, Pearson’s chi-squared test for gender and site of onset and the Kolmogorov–Smirnov test for the age at onset. The *Q*-test power calculation was made using Stata 15 (Stata Corp, College Station, TX, USA). All other analyses were done in R 3.4.4 using the Metabin function of the Meta library [30]. The *p-*value threshold used in this study to indicate statistical significance was 0.05.

### Alzheimer’s Disease Neuroimaging Initiative

Data used in the preparation of this article were obtained from the Alzheimer’s Disease Neuroimaging Initiative (ADNI) database (adni.loni.usc.edu). The ADNI was launched in 2003 as a public-private partnership, led by Principal Investigator Michael W. Weiner, MD. The primary goal of ADNI has been to test whether serial magnetic resonance imaging (MRI), positron emission tomography (PET), other biological markers, and clinical and neuropsychological assessment can be combined to measure the progression of mild cognitive impairment (MCI) and early Alzheimer’s disease (AD).

## Results

### *C9orf72* repeat expansion distribution in the new cohort

For the British dataset, the median age of ALS onset was 62.4 years and for controls, 60.1 years, with a male-female ratio of 62:38. In the ADNI dataset the median age was 73.2 and the male-female ratio 49:51.

We identified no difference in the distributions of repeat size between ALS and controls in the 1–23 repeat interval (Kolmogorov-Smirnov test, *p*-value = 0.96). Nine cases and one control were found to carry 24–30 repeats. Five controls were found to carry expansions whose length is rarely seen in unaffected controls and among which a few were hundreds of repeats long (Fig. 1 and Additional file 1: **Table S1**). Specifically, five British controls carried large expansions up to hundreds of repeats (sizes 60, 357, 406, 413 and 484) and one British control carried an intermediate repeat expansion of 26 repeats. 6.6% of cases had a repeat of size > 30.

### Meta-analysis of the 24–30 repeat expansion

A literature review [17] identified four studies which provided original data on the number of patients carrying 24–30 repeats, including one study from North America [23], one study from Italy [16], one study from France [15], and one study from Spain [13]. We extended the review to all articles published up to January 2019 and identified one further study [28] providing data suitable for our meta-analysis. However, in this additional study, most of the ALS cases overlapped with our British dataset, and we therefore excluded it from our analysis. Therefore, after reproducing the previously published meta-analysis, we performed two new meta-analyses. In the first we added our British samples (1295 cases and 340 controls) and in the second we added our British/ADNI dataset (1295 cases and 613 controls). The final dataset used in this study comprised 5071 cases and 3747 controls. We were able to reproduce the association between the *C9orf72* 24–30 repeat expansion and ALS using the previously used four datasets (fixed-effect model odds ratio = 5.13, 95% confidence interval = 1.28–22.24, *p*-value = 0.03) (Table 1A). The association was replicated after including our cohorts, both when the British cohort was added (fixed-effect model odds ratio = 4.01, 95% confidence interval = 1.21–13.26 *p*-value = 0.02) (Table 1B) and when the British/ADNI dataset was added (fixed-effect model odds ratio = 4.82, 95% confidence interval = 1.45–15.96, *p*-value = 0.01) (Table 1C). The use of the fixed-effect model was suggested by the chi-square *Q*-test which did not show heterogeneity (*p*-value = 0.61), but this was underpowered to detect heterogeneity (power = 12.07%), suggesting that a random-effects model is a more appropriate model to use. Under a random-effects model, we did not show association using the four studies from the previous meta-analysis only (*p*-value = 0.07). Using the UK/ADNI dataset we were able to show this association under the random-effects model hypothesis also (odds ratio = 4.2, 95% confidence interval = 1.23–14.35, *p*-value = 0.02) (Table 1C).

**Table 1:** Meta-analyses on the influence of *C9orf72* 24–30 repeat-long expansions on the risk of ALS. (A) Replication of the previously published meta-analysis (Y. Chen et al.). (B) Adding our British dataset to the 4 used in Y. Chen et al. (C) Adding our British/ADNI dataset to the 4 used in Y. Chen et al

	N. of samples 24–30 repeats Cases/Controls	Total N. of samples Cases/Controls	OR	95% Conf. Interval		% Weight Fixed/Random
A - Y. Chen et al. meta-analysis datasets						
Garcia-Redondo et al.	1/0	781/248	0.95	0.04	23.52	32.8/22.7
Ratti et al.	1/0	1275/862	2.03	0.08	49.89	25.8/22.7
Rutherford et al.	3/0	995/1444	10.19	0.53	197.45	17.6/26.5
Millecamps et al.	6/0	725/580	10.19	0.59	186.57	23.8/28.1
M-H pooled (fixed-effect)	11/0	3776/3134	5.13	1.18	22.24	100.0
M-H pooled (random-effects)	11/0	3776/3134	4.16	0.90	19.14	100.0
Heterogeneity chi-squared Q = 1.82 (d.f. = 3) *p* = 0.61, power = 12.07%						
I-squared (variation in OR attributable to heterogeneity) = 0.0% (0.0–74.8%)						
Fixed-effect Test of OR = 5.13: z = 2.18 *p* = 0.03						
Random-effects Test of OR = 4.16: z = 1.83 *p* = 0.07						
B - Y. Chen et al. meta-analysis datasets + our British dataset						
Garcia-Redondo et al.	1/0	781/248	0.95	0.04	23.52	19.5/14.7
Ratti et al.	1/0	1275/862	2.03	0.08	49.89	15.3/14.7
Rutherford et al.	3/0	995/1444	10.19	0.53	197.45	10.5/17.2
Millecamps et al.	6/0	725/580	10.49	0.59	186.57	14.2/18.2
British dataset	9/1	1295/340	2.37	0.30	18.79	40.5/35.2
M-H pooled (fixed-effect)	20/1	5071/3474	4.01	1.21	13.26	100.0
M-H pooled (random-effects)	20/1	5071/3474	3.41	1.00	11.66	100.0
Heterogeneity chi-squared Q = 2.00 (d.f. = 4), *p* = 0.74, power = 12.83%						
I-squared (variation in OR attributable to heterogeneity) = 0.0% (0.0–58.4%						
Fixed-effect Test of OR = 4.01: z = 2.28 *p* = 0.02						
Random-effects Test of OR = 3.41: z = 1.96 *p* = 0.05						
C - Y. Chen et al. meta-analysis datasets + our British/ADNI dataset						
Garcia-Redondo et al.	1/0	781/248	0.95	0.04	23.52	20.7/14.7
Ratti et al.	1/0	1275/862	2.03	0.08	49.89	16.3/14.7
Rutherford et al.	3/0	995/1444	10.19	0.53	197.45	11.1/17.2
Millecamps et al.	6/0	725/580	10.49	0.59	186.57	15.0/18.2
British/ADNI dataset	9/1	1295/613	4.28	0.54	33.88	36.9/35.2
M-H pooled (fixed-effect)	20/1	5071/3747	4.82	1.45	15.96	100.0
M-H pooled (random-effects)	20/1	5071/3747	4.20	1.23	14.35	100.0
Heterogeneity chi-squared Q = 1.80 (d.f. = 4), *p* = 0.77, power = 13.01%						
I-squared (variation in OR attributable to heterogeneity) = 0.0% (0.0–53.7%)						
Fixed-effect Test of OR = 4.82: z = 2.57 *p* = 0.01						
Random-effects Test of OR = 4.20: z = 2.29 p = 0.02						

No evidence of publication bias from either the Egger (*p*-value = 0.69) or Begg (*p*-value = 0.14) test was observed (Additional file 1: **Figure S1**). Sensitivity testing, recalculating the pooled odds ratios by omitting one study per iteration (Additional file 1: **Table S2**) showed that results were consistent, without major fluctuations.

### Association between *C9orf72* intermediate expansion on the aggregated dataset

We also investigated the association between the intermediate repeats and ALS in a final dataset generated from the aggregation of the 5 datasets used for the meta-analysis (5071 cases and 3747 controls). The association was demonstrated both with the chi-squared test (*p*-value = 5 × 10^− 4^, odds ratio = 14.83, 95% confidence interval 1.99–110.57) and the Fisher’s exact test (*p*-value = 2 × 10^− 4^). Considering that the frequency of the *C9orf72* expanded repeat greatly varies across European populations [5, 7, 13–15, 31], we attempted to investigate whether this is also true for the intermediate repeats. To do so we split the aggregated dataset cases in two groups, 3015 northern European patients (Americans, French and British) and 2056 southern European patients (Italians and Spanish). Both the chi-squared test (*p*-value = 5 × 10^− 3^, odds ratio = 6.17, 95% confidence interval 1.43–26.61) and the Fisher’s exact test (*p*-value = 5 × 10^− 3^) showed a significant difference between the two groups. On the basis of intermediate repeat heterogeneity between the two groups, we tested the association between the intermediate repeats and ALS in the two groups separately. For the northern European dataset (3015 cases and 2637 controls) the resulting odds ratio was 15.83 (95% confidence interval 2.11–118.67, chi-squared *p*-value = 3 × 10^− 4^ and Fisher’s exact test *p*-value = 10^− 4^). In the southern European dataset (2056 cases and 1110 controls) the association was not significant (Fisher’s exact test *p*-value = 0.54). However, the low frequency of intermediate expansions (< 0.1% in cases and not present in controls) limits the statistical power to conclude if there is an association in this group (power < 10%).

### Clinical phenotype analysis

For our 1295 British patients, we compared the age at onset, time from first symptom to death (or last visit for censored cases) and site of onset (bulbar or spinal), between the patients who carried a non-expanded number of repeats (< 24 in 1201 patients), an intermediate number of repeats (> 23 and < = 30 in 9 patients) and a full expansion (> 30 in 85 patients). We found no significant differences between the intermediate repeat carriers and the other patients. Consistent with previous findings [11, 32–34], patients carrying a full expansion had a shorter survival time (*p*-value < 0.0001, median = 2.49 years and 25–75% quantiles = 1.88–3.71 years) and a younger age at onset (*p*-value = 0.009, mean = 58.39 ± 8.46 years) compared to patients carrying a non-expanded number of repeats (survival median = 3.01 years and 25–75% quantiles = 2.10–4.55 years, mean age at onset = 60.90 ± 11.13 years). No difference in terms of gender prevalence and site of onset were observed between the three groups. A demographic table for the three groups is available in the Supplementary material (Additional file 1: **Table S2**).

## Discussion

We have shown that intermediate repeat expansions in the *C9orf72* gene are associated with ALS. An expanded hexanucleotide repeat in *C9orf72* is the most common cause of ALS. While expansions of hundreds of repeats in size are clearly associated with the disease, a lower limit has not yet been identified. A few studies have suggested that 24–30 repeats might be sufficient to cause the disease [12, 17]. However, considering that the statistical power of the datasets used was limited, further investigation was essential. We have added 1295 cases and 613 controls to the meta-analysis and demonstrated the association of such intermediate expansions with ALS both under a fixed-effect (*p*-value = 0.01) and a random-effects model (*p*-value = 0.02). The latter tells us that the association between 24 and 30 repeats and the disease exists without assuming that the effect of this mutation is homogenous across the patient groups. This up-to-date figure obtained from a meta-analysis on 5071 ALS patients and 3747 controls from 5 populations (UK, USA, Spain, Italy and France) provides definitive evidence for the association between intermediate repeats and ALS (*p*-value = 2 × 10^− 4^) and expands the role of *C9orf72* in the development of ALS by enlarging the number of cases that can be explained, with direct implications for research and clinical practice. Indeed, we suggest the threshold to discriminate between neutral and pathogenic *C9orf72* expansions should be lowered to 23, considering as pathogenic, expansions of 24 or more repeats, in agreement with previously reported observations [5, 17].

Expansions of 20–23 repeats have contradictory evidence of association with ALS. This suggests that if such an association exists, their contribution to disease risk is weaker than for longer expansions, and we need larger datasets to investigate it. Due to the very low frequency of intermediate repeats, further investigation and larger sample sizes are needed to consolidate our results and determine whether to lower the threshold further. International initiatives such as Project MinE, whose aim is to collect genetic data (including the length of the *C9orf72* repeat) of over 10,000 thousand people with ALS, will be crucial to this end. Interestingly, we also observed a few controls with very large expansions that are only rarely observed in non-affected individuals. We have openly released the repeat frequency in our cohort for a wide range of lengths, to allow further analysis when new cohorts become available (Additional file 1: **Table S1**). Finally, we looked at age at onset, survival time, gender prevalence and site of onset of the intermediate repeat carriers, and we found no significant differences from the other patients. However, this analysis was limited by their small number (9), which does not allow us to make any conclusion about the clinical characterization of this sub-group of patients.

## Additional file

Additional file 1: **Figure S1.** Bias detection in the meta-analysis: A) Egger test; B) Begg test. C) Funnel plot of the 5 studies used in our meta analysis. **Table S1.** *C9orf72* expansion analysis results obtained with ExpansionHunter on the British/ADNI dataset. **Table S2.** Pooled odds ratios by omitting one study per iteration. Demographic table. (DOCX 3047 kb)
