# Supplementary material for: C9orf72 intermediate expansions of 24–30 repeats are associated with ALS
Source: Acta Neuropathol Commun. 2019 Jul 17;7:115. doi: 10.1186/s40478-019-0724-4 (PMC6637621; doi:10.1186/s40478-019-0724-4)
Supplement: Supplementary file 1 — Figure S1. Bias detection in the meta-analysis: A) Egger test; B) Begg test. C) Funnel plot of the 5 studies used in our meta analysis. Table S1. C9orf72 expansion analysis results obtained with ExpansionHunter on the British/ADNI dataset. Table S2. Pooled odds ratios by omitting one study per iteration. Demographic table. (DOCX 3047 kb) [file 40478_2019_724_MOESM1_ESM.docx]

***C9orf72* intermediate expansions of 24–30 repeats are associated with ALS supplementary material**

Alfredo Iacoangeli^a,b^**^*^**, Ahmad Al Khleifat^b^, Ashley R Jones^b^, William Sproviero^b^, Aleksey Shatunov^b^, Sarah Opie-Martin^b^, Karen E Morrison^c^, Pamela J Shaw^d^, Isabella Fogh^b,e^, Christopher E Shaw^b,^^f^, Richard J Dobson^a,g,h^, Stephen J Newhouse^a,g,h^, Ammar Al-Chalabi^b,i^

^a^Department of Biostatistics and Health Informatics, King’s College London, London, UK; ^b^Maurice Wohl Clinical Neuroscience Institute, King’s College London, Department of Basic and Clinical Neuroscience, London, UK; ^c^Faculty of Medicine, University of Southampton, University Hospital Southampton, UK; ^d^Sheffield Institute for Translational Neuroscience, University of Sheffield, UK; ^e^Department of Neurology and Laboratory of Neuroscience, IRCCS Istituto Auxologico Italiano, Milan, Italy; ^f^UK Dementia Research Institute, King’s College London, UK; ^g^Farr Institute of Health Informatics Research, UCL Institute of Health Informatics, University College London, London, UK; ^h^National Institute for Health Research (NIHR) Biomedical Research Centre and Dementia Unit at South London and Maudsley NHS Foundation Trust and King’s College London London, UK; ^i^King's College Hospital, Bessemer Road, London, SE5 9RS, UK.

Additional file 1: Figure S1 – Bias detection in the meta-analysis: A) Egger test; B) Begg test. C) Funnel plot of the 5 studies used in our meta analysis.

Additional file 1: Table S2 – Pooled odds ratios by omitting one study per iteration.

|  | N. of ALS cases | N. of UK controls | N. of ADNI controls | N. of ALS cases | N. of UK controls | N. of ADNI controls |
| --- | --- | --- | --- | --- | --- | --- |
| Total N. of samples | 1295 | 340 | 273 | 1295 | 340 | 273 |
| N. repeats | N. of samples carrying at least one allele of N. repeats | | | N. of observed alleles of N. repeats | | |
| 2 | 974 | 269 | 205 | 1297 | 349 | 283 |
| 3 | 1 | 1 | 3 | 1 | 1 | 3 |
| 4 | 62 | 14 | 13 | 62 | 14 | 14 |
| 5 | 350 | 106 | 80 | 383 | 112 | 89 |
| 6 | 137 | 34 | 35 | 139 | 35 | 38 |
| 7 | 34 | 14 | 7 | 36 | 14 | 8 |
| 8 | 303 | 77 | 58 | 318 | 80 | 60 |
| 9 | 15 | 7 | 6 | 15 | 7 | 6 |
| 10 | 79 | 23 | 15 | 79 | 23 | 15 |
| 11 | 47 | 14 | 6 | 48 | 14 | 6 |
| 12 | 23 | 4 | 8 | 23 | 4 | 8 |
| 13 | 27 | 9 | 1 | 27 | 9 | 1 |
| 14 | 23 | 5 | 2 | 23 | 5 | 2 |
| 15 | 8 | 4 | 0 | 8 | 4 | 0 |
| 16 | 11 | 2 | 2 | 11 | 2 | 2 |
| 17 | 5 | 0 | 2 | 5 | 0 | 2 |
| 18 | 8 | 0 | 3 | 8 | 0 | 3 |
| 19 | 5 | 0 | 3 | 5 | 0 | 3 |
| 20 | 4 | 1 | 2 | 4 | 1 | 2 |
| 21 | 2 | 0 | 0 | 2 | 0 | 0 |
| 22 | 0 | 0 | 1 | 0 | 0 | 1 |
| 23 | 1 | 0 | 0 | 1 | 0 | 0 |
| 24 | 0 | 0 | 0 | 0 | 0 | 0 |
| 25 | 0 | 0 | 0 | 0 | 0 | 0 |
| 26 | 4 | 1 | 0 | 4 | 1 | 0 |
| 27 | 2 | 0 | 0 | 2 | 0 | 0 |
| 28 | 2 | 0 | 0 | 2 | 0 | 0 |
| 29 | 0 | 0 | 0 | 0 | 0 | 0 |
| 30 | 1 | 0 | 0 | 1 | 0 | 0 |
| 31+ | 85 | 5 | 0 | 86 | 5 | 0 |

Additional file 1: Table S1 - *C9orf72* expansion analysis results obtained with ExpansionHunter on the British/ADNI dataset.

| Expansion  length | Number of patients | Male/female  ratio | Censored/dead ratio | Age at onset mean  (years) | Survival median  (years) | Survival  quantiles (25%/75%) | Bulbar/spinal ratio |
| --- | --- | --- | --- | --- | --- | --- | --- |
| Non-expanded | 1,201 | 1.65 | 0.083 | 60.90±11.13 | 3.01 | 2.10/4.55 | 0.37 |
| Intermediate | 9 | 2 | 0.125 | 63.48±11.17 | 3.02 | 2.44/3.68 | 0.5 |
| Full expansion | 85 | 1.18 | 0.014 | 58.39±8.46 | 2.49 | 1.88/3.71 | 0.5 |

Table S2 – Demographic table.
